# The Effects of Propofol Cardioplegia on Blood and Myocardial Biomarkers of Stress and Injury in Patients With Isolated Coronary Artery Bypass Grafting or Aortic Valve Replacement Using Cardiopulmonary Bypass: Protocol for a Single-Center Randomized Controlled Trial

**DOI:** 10.2196/resprot.3353

**Published:** 2014-07-08

**Authors:** Zoe E Plummer, Sarah Baos, Chris A Rogers, M-Saadeh Suleiman, Alan J Bryan, Gianni D Angelini, James Hillier, Richard Downes, Eamonn Nicholson, Barnaby C Reeves

**Affiliations:** ^1^Clinical Trials and Evaluation UnitUniversity of BristolBristolUnited Kingdom; ^2^Bristol Heart InstituteUniversity of BristolBristolUnited Kingdom; ^3^Division of Specialised ServicesUniversity Hospitals Bristol NHS Foundation TrustBristolUnited Kingdom

**Keywords:** cardiac surgery, anesthetics, cardiopulmonary bypass, ischemia, reperfusion, cardioplegia, aortic valve, coronary artery, troponin, clinical trials, randomized

## Abstract

**Background:**

Despite improved myocardial protection strategies, cardioplegic arrest and ischemia still result in reperfusion injury. We have previously published a study describing the effects of propofol (an anesthetic agent commonly used in cardiac surgery) on metabolic stress, cardiac function, and injury in a clinically relevant animal model. We concluded that cardioplegia supplementation with propofol at a concentration relevant to the human clinical setting resulted in improved hemodynamic function, reduced oxidative stress, and reduced reperfusion injury when compared to standard cardioplegia.

**Objective:**

The Propofol cardioplegia for Myocardial Protection Trial (ProMPT) aims to translate the successful animal intervention to the human clinical setting. We aim to test the hypothesis that supplementation of the cardioplegic solution with propofol will be cardioprotective for patients undergoing isolated coronary artery bypass graft or aortic valve replacement surgery with cardiopulmonary bypass.

**Methods:**

The trial is a single-center, placebo-controlled, randomized trial with blinding of participants, health care staff, and the research team. Patients aged between 18 and 80 years undergoing nonemergency isolated coronary artery bypass graft or aortic valve replacement surgery with cardiopulmonary bypass at the Bristol Heart Institute are being invited to participate. Participants are randomly assigned in a 1:1 ratio to either cardioplegia supplementation with propofol (intervention) or cardioplegia supplementation with intralipid (placebo) using a secure, concealed, Internet-based randomization system. Randomization is stratified by operation type and minimized by diabetes mellitus status. Biomarkers of cardiac injury and metabolism are being assessed to investigate any cardioprotection conferred. The primary outcome is myocardial injury, studied by measuring myocardial troponin T. The trial is designed to test hypotheses about the superiority of the intervention within each surgical stratum. The sample size of 96 participants has been chosen to achieve 80% power to detect standardized differences of 0.5 at a significance level of 5% (2-tailed) assuming equal numbers in each surgical stratum.

**Results:**

A total of 96 patients have been successfully recruited over a 2-year period. Results are to be published in late 2014.

**Conclusions:**

Designing a practicable method for delivering a potentially protective dose of propofol to the heart during cardiac surgery was challenging. If our approach confirms the potential of propofol to reduce damage during cardiac surgery, we plan to design a larger multicenter trial to detect differences in clinical outcomes.

**Trial Registration:**

International Standard Randomized Controlled Trial Number (ISRCTN): 84968882; http://www.controlled-trials.com/ISRCTN84968882/ProMPT (Archived by WebCite at http://www.webcitation.org/6Qi8A51BS).

## Introduction

During cardiac surgery with cardiopulmonary bypass (CPB), a cardioplegia (heart-stopping) solution is used to arrest the heart. Although beneficial for the surgical procedure, the oxygen/nutrient deficit and restriction in blood supply (ischemia) can result in myocardial damage and dysfunction. In addition, restoration of oxygenated blood flow (reperfusion) after a period of ischemia can cause further (and often more severe) damage. This is known as ischemia/reperfusion (I/R) injury.

Loss of control over cellular calcium mobilization and the generation of reactive oxygen species (ROS) are known to be key events critical to the induction of I/R damage [1]. Elevated intracellular calcium leads to the destruction of mitochondrial cell membrane integrity [2] and eventual recruitment of macrophages and neutrophils to the area causing further damage to surrounding tissue. There are several sources of ROS generation with all species interacting with numerous cellular targets. ROS attack a wide range of biological molecules resulting in deleterious wide-ranging effects, including attack of the cardiomyocyte [3]. Furthermore, cytosolic calcium loading and the generation of ROS can result in the opening of the mitochondrial permeability transition pore (MPTP). Mitochondrial disruption consequently leads to cardiomyocyte death [1,4].

Strategies to protect the heart during cardiac surgery include interventions that target the mitochondria, such as alteration of cardioplegia temperature, method of delivery and composition, and the use of calcium transport modulators and/or inhibitors of the MPTP [1,5-11]. A number of anesthetic agents have also been implicated in cardioprotection strategies [12-14]. Inhalation anesthetics have been shown to decrease myocardial oxygen demand and contractility [15] and intravenous anesthetics have been shown to exhibit antioxidant effects [16]. Both are reported to play a role in the reduction of the systemic anti-inflammatory response.

Propofol is a general anesthetic widely used for the induction and maintenance of anesthesia during cardiac surgery and for postoperative sedation. In addition to its anesthetic effect, propofol has been reported to confer protection against damage to the myocardium during oxidative stress [17] and reperfusion [18,19]. Various mechanisms have been proposed to explain this cardioprotective effect, including inhibition of plasma membrane calcium channels [20,21], free radical scavenging [22-25], and enhancing antioxidant capacity [26,27]. Furthermore, reports have shown propofol can inhibit the MPTP in isolated mitochondria [28,29], although nonclinical concentrations were employed in these studies.

We have previously published a study describing the effects of propofol on metabolic stress, cardiac function, and injury in a clinically relevant animal model of normothermic cardioplegic arrest and CPB [30]. We were able to conclude that cardioplegia supplementation with propofol at a concentration relevant to the human clinical setting resulted in improved hemodynamic function, reduced oxidative stress, and reduced I/R injury when compared to standard cardioplegia.

The aim of our current trial is to translate propofol supplementation from our animal model to a human clinical setting by investigating the benefits of using propofol as an adjunct to cardioplegia for patients undergoing coronary artery bypass grafting (CABG) or aortic valve replacement (AVR) surgery using CPB. Specific objectives are:

1. To estimate mean differences in biomarkers of cardiac injury and metabolism between groups of participants having isolated CABG with CPB using warm blood cardioplegia with propofol supplementation vs supplementation with placebo.
2. To estimate mean differences in biomarkers of cardiac injury and metabolism between groups of participants having AVR with CPB using cold blood cardioplegia with propofol supplementation vs supplementation with placebo (intralipid).

## Methods

### Overview

The Propofol cardioplegia for Myocardial Protection Trial (ProMPT) is designed as a single-center, placebo-controlled, randomized trial. Participants, health care staff, and members of the research team involved in data collection or providing health care, except for the perfusionist, are blinded to a participant’s allocation.

### Trial Population and Recruitment Procedure

Patients undergoing nonemergency isolated CABG or AVR with CPB at the Bristol Heart Institute (BHI) are being invited to participate.

Participants may enter the trial if they are male or female, between 18 and 80 years, having elective or urgent CABG or AVR with CPB, and are able to give full informed consent for the trial. Patients may not enter the trial if any of the following apply: (1) previous cardiac surgery, (2) combined CABG and AVR, (3) emergency or salvage operation, (4) chronic renal failure requiring dialysis, (5) current congestive heart failure, (6) left ventricular (LV) ejection fraction <30% (ie, poor LV function), (7) allergy to peanuts, eggs, egg products, soybeans, or soy products (these are intralipid ingredients), or (8) participating in another interventional trial.

Potential trial participants are identified from operating theater lists and are sent or handed an invitation letter and information leaflet that has been approved by a NHS Research Ethics Committee (REC). They have at least 24 hours to consider whether to participate. If there is insufficient time to consider taking part, a potential participant is not approached to ask for written informed consent.

After admission to the BHI, patients are seen by a member of the research team who answers any questions, confirms eligibility, and requests and witnesses written informed consent. Details of all patients approached for the trial and reasons for nonparticipation (eg, reason for being ineligible or declining consent) are being documented.

### Randomization

Each participant is randomly assigned in a 1:1 ratio within CABG or AVR surgery stratum to standard cardioplegia with either propofol (intervention) or intralipid (placebo) supplementation using a secure, concealed, central Internet-based randomization system (Sealed Envelope Ltd). Standard cardioplegia is warm blood for CABG and cold blood for AVR. Randomization is minimized by diabetes mellitus status, defined as requirement for oral or insulin medication to control blood sugar at the time of admission. A member of the Clinical Trials and Evaluation Unit (CTEU) Bristol who is not involved in data collection or providing health care randomizes a participant shortly before surgery and communicates the allocation to the perfusionist. If a participant is unexpectedly rescheduled, the trial number and randomized allocation is retained.

### Intervention and Placebo

#### Overview

Propofol (Fresenius Propoven 1%) is licensed for the induction and maintenance of anesthesia and is used for these purposes by most cardiac anesthetists at the BHI.

Intralipid emulsion is used as the vehicle for propofol administration; therefore, they have exactly the same consistency and milky appearance. It has been suggested that intralipid is itself cardioprotective (based on recovery of cardiac function in rats) [31], but it does not appear to confer protection against cardiac injury [32]. We wish to test the specific hypothesis that propofol supplementation is cardioprotective and a recognized mechanism is postulated. For these reasons, intralipid has been chosen as the placebo for this trial.

Propofol is diluted in a 1:5 ratio with 0.9% sodium chloride as recommended by the manufacturer to achieve a working solution of 2000 μg/mL. Intralipid is diluted in the same manner.

Propofol and intralipid for use in the trial are labeled and dispensed by the Bristol Royal Infirmary (BRI) pharmacy in accordance with Good Clinical Practice (GCP).

#### Standard Cardioplegia Composition and Delivery

Calafiore warm blood cardioplegia (for CABG): A 60 mL syringe is prepared with 20 mL of 15% potassium chloride (2 mmol K^+^/mL) and 5 mL of 50% magnesium sulfate (2 mmol Mg^2+^/mL), resulting in a ratio of 4:1 potassium:magnesium in the syringe driver. A roller pump draws oxygenated blood from the oxygenator and the potassium–magnesium mixture is added by syringe pump downstream. Intermittent antegrade delivery is used according to local protocol.

Cold blood cardioplegia (for AVR): A 500 mL prebagged solution of Harefield Hospital Formulation (IVEX Pharmaceuticals Ltd, Larne, Northern Ireland, UK) is used. A roller pump draws up oxygenated blood from the oxygenator and the cardioplegia solution is added in a 4:1 blood:cardioplegia ratio. Cold cardioplegia is given at a temperature of approximately 4°C and by either antegrade or retrograde delivery (or a mixture of both) according to local protocol.

#### Anesthetic Management and Supplementation of Cardioplegia

Anesthetic management adheres strictly to a locally agreed protocol (see Multimedia Appendix 1) and all other aspects of the patient’s preoperative and postoperative management are in accordance with existing protocols in use in the BHI.

**Figure 1:**
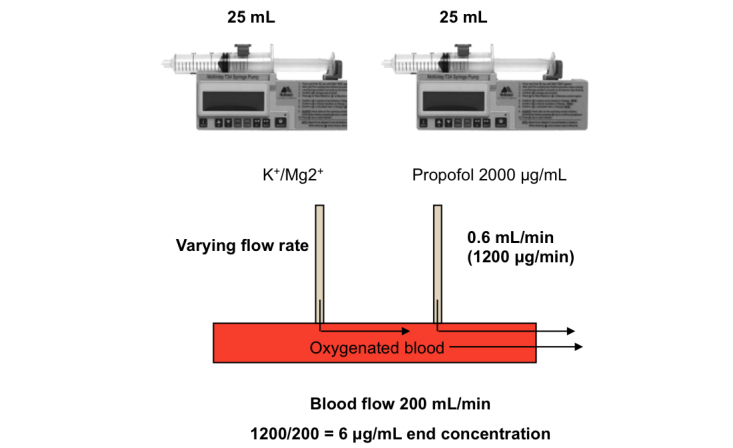
CABG warm blood cardioplegia. Propofol is first diluted from 10,000 μg/mL to 2000 μg/mL using 0.9% sodium chloride. The diluted propofol solution is added to the cardioplegia by attaching an additional syringe pump to the line downstream of the blood oxygenator. This method is identical to that used for adding potassium and magnesium to the oxygenated blood. The syringe driver is set to 0.6 mL/min resulting in a 6 μg/mL supplementation of the blood/cardioplegia mix during delivery. For the placebo group, cardioplegia is supplemented with placebo in the same manner as described for propofol. In the event addition of propofol is indicated, this is substituted with an equivalent volume of intralipid.

**Figure 2:**
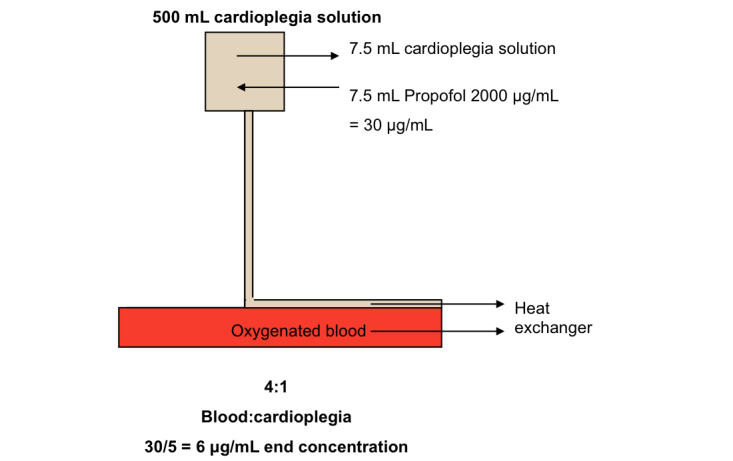
AVR cold blood cardioplegia. Propofol is first diluted from 10,000 μg/mL to 2000 μg/mL using 0.9% sodium chloride. Diluted propofol is added directly to a 500 mL bag of 4:1 cardioplegia solution by 1:1 (vol:vol) substitution. For the placebo group, cardioplegia is supplemented with placebo in exactly the same manner as described for propofol. In the event addition of propofol is indicated, this is substituted with an equivalent volume of intralipid.

For CABG procedures, propofol (or intralipid) is added to the cardioplegia by attaching an additional syringe pump to the line, downstream of the blood oxygenator (Figure 1). For AVR procedures, propofol (or an equivalent volume intralipid) is added directly to a 500 mL bag of 4:1 cardioplegia solution (Figure 2).

For both intervention groups, the cardioplegia solution (blood–cardioplegia mix) has a final concentration target of 6 μg/mL of supplemented propofol. This concentration is significantly below the level routinely observed in the circulation during induction of anesthesia for cardiac surgery, but higher than during maintenance of anesthesia when cardioplegia is administered (ie, a concentration to which the myocardium is routinely exposed).

### Primary Outcome

**Figure 3:**
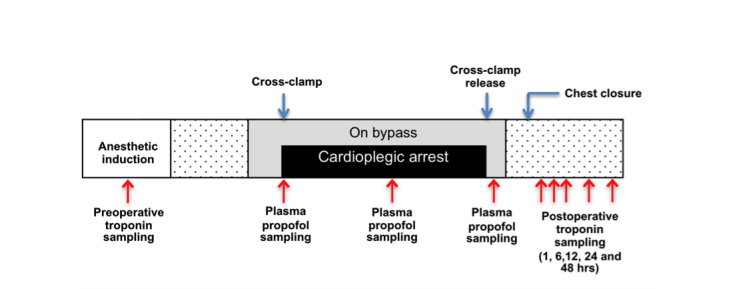
Measurement of myocardial injury and propofol concentration. The primary outcome is myocardial injury, assessed by measuring myocardial troponin T in serum from blood samples collected preoperatively and at 1, 6, 12, 24, and 48 hours after chest closure. The concentration of plasma propofol is measured in blood samples collected from the cardioplegia/bypass circuit immediately before aortic cross-clamping, once during cardioplegia (after blood–cardioplegia mixing), and 10 min post cross-clamp release.

The primary outcome is myocardial injury, assessed by measuring myocardial troponin T in serum from blood samples collected preoperatively and at 1, 6, 12, 24, and 48 hours after chest closure (Figure 3). Although absolute times of sampling after reperfusion could vary between participants, blinding of the intervention prevents any systematic difference between groups.

### Secondary Outcomes

Data are collected to characterize the following secondary outcomes:

1. Myocardial ischemic stress assessed using biopsies taken from left and right ventricles immediately before aortic cross-clamping and 10 minutes after cross-clamp release. Gene expression and cellular changes associated with stress and injury-signaling pathways are to be studied in a small subset of biopsies using metabolite/RNA extracts.
2. Systemic metabolic stress assessed by measuring lactate in blood samples collected preoperatively and 1, 6, 12, 24, and 48 hours after chest closure.
3. Blood pH measured by using the blood samples collected preoperatively and 1, 6, 12, 24, and 48 hours after chest closure.
4. Renal function assessed by measuring creatinine in serum from blood samples collected preoperatively and 1, 6, 12, 24, and 48 hours after chest closure.
5. The concentration of plasma propofol measured in blood samples collected immediately before aortic cross-clamping, once during cardioplegia (after blood–cardioplegia mixing), and 10 minutes after cross-clamp release (Figure 3). Blood is taken from the cardioplegia/bypass circuit.
6. Length of intensive care unit (ICU) / high-dependency unit (HDU) stay.
7. Clinical outcomes and serious adverse events (SAEs), such as serious postoperative morbidity (eg, myocardial infarction, permanent stroke, renal failure defined as new need for hemodialysis) and death from any cause. Case report forms used for data collection specify 26 SAEs that are anticipated after cardiac surgery.
8. Patient health status monitored using customized questionnaires administered preoperatively and completed by participants as a postal questionnaire 3 months postoperatively. Also, CABG and AVR patients are asked to complete the Coronary Revascularisation Outcome Questionnaire (CROQ) [33] and the Minnesota Living with Heart Failure (MLHF) Questionnaire [34], respectively. The EQ-5D health questionnaire is administered preoperatively and all participants complete this again at 3 months as part of the postal questionnaire.

The primary outcome is myocardial injury, assessed by measuring myocardial troponin T in serum from blood samples collected preoperatively and at 1, 6, 12, 24, and 48 hours after chest closure. The concentration of plasma propofol is measured in blood samples collected from the cardioplegia/bypass circuit immediately before aortic cross-clamping, once during cardioplegia (after blood–cardioplegia mixing), and 10 minutes after cross-clamp release.

### Sample Size

The trial is designed to test hypotheses about the superiority of the intervention.

A sample size of 96 has been chosen to enable the trial to detect a standardized difference of 0.5 between the propofol-supplemented and placebo groups, with 80% power at a significance level of 5% (2-tailed) within each surgical stratum. The target difference of 0.5 standard deviations is of moderate magnitude [35], is consistent with our experience of many of these outcomes in previous research to evaluate other cardiac surgery interventions, and is appropriate for an early phase trial. Estimation of the power of the trial assumed that the biomarkers, including the primary outcome, are measured at baseline and 5 times after the intervention and that the correlations between outcomes measured at baseline and after intervening and between postintervention outcomes measured on multiple occasions are 0.5.

### Statistical Analysis

Analyses will be based on the intention-to-treat principle, and will use data from all patients randomized (ie, the analyses will use the complete dataset). Continuous outcomes will be analyzed by regression modeling, transforming data logarithmically if required and adjusting for baseline values where available, and using mixed models for repeated measures. Mixed models allow all patients with data to be included in the analysis; that is, partial missing data (assumed missing at random) is permitted. Prerandomization and subsequent values will be modeled jointly, in preference to the prerandomization value being modeled as a covariate, to avoid the need either to exclude cases with missing preoperative measures or to impute missing preoperative values. Interactions between treatment and time will be examined and, if significant at the 5% level, results will be reported separately for each postoperative time point; otherwise, an overall treatment effect will be reported. Findings will be reported as effect sizes with 95% confidence intervals. Time in ICU/HDU will be analyzed as time-to-event data using Cox regression modeling.

The trial is not powered to detect differences in clinical outcomes and their frequencies will be tabulated descriptively in accordance with guidelines for reporting randomized controlled trials (RCTs) [36].

We do not have a prior expectation that the effect of propofol supplementation will differ by operation, but we will test for this possibility by fitting a treatment by surgery type interaction. If the interaction is statistically significant, surgery-specific effects will be reported along with the results of the interaction test. If, as anticipated, the interaction is not statistically significant at the 10% level, the overall treatment effect will be reported. Similarly, we will test the interaction of propofol supplementation with diabetic status and effects specific to diabetic status will be estimated if this interaction reaches 10% statistical significance.

The primary analysis will take place when follow-up is complete for all recruited patients. No interim analysis is planned.

### Ethical Approval and Clinical Trial Authorization

The West Midlands REC approved the trial protocol on the November 16, 2009. The Medicines and Healthcare products Regulatory Agency (MHRA) granted clinical trial authorization on December 11, 2009.

### Adverse Events

Adverse events (AEs) will be recorded and reported according to University Hospitals Bristol NHS Foundation Trust and MHRA guidelines. In cardiac surgery, postoperative transient complications are expected and are not infrequent. The research team is required to notify the sponsor about deaths and unexpected nonfatal SAEs. Unexpected events are those not listed in the trial protocol or on the case report forms. The sponsor will inform the research team which SAEs should be reported to the REC and/or MHRA. Data on AEs are being collected from randomization for the duration of the participant’s postoperative hospital stay and for the 3-month follow-up period.

### Measures to Reduce the Risk of Bias

The trial includes several features designed to minimize the risk of bias. Concealed randomization will prevent selection bias. Blinding of the research team, clinical staff responsible for caring for patients (surgeons, anesthetists, and nurses), and participants will minimize performance and detection biases. Moreover, outcome measures have been defined as far as possible on the basis of objective criteria. An independent laboratory technician, without knowledge of treatment allocation, will measure biochemical markers.

The patient information leaflet and the process of obtaining informed consent describes the uncertainty about the effects of cardioplegia supplementation with propofol. Therefore, in the event of inadvertent unblinding of a participant, he or she should not have a strong expectation that either method should lead to a more favorable result.

The trial will be analyzed on an intention-to-treat basis (ie, outcomes will be analyzed according to the treatment allocation), irrespective of future management and events, and every effort will be made to include all randomized patients. The fact that trial recruitment is from a single center and coordination is by a UK Clinical Research Collaboration (UKCRC)-registered trials unit on site should promote the completeness of follow-up. Blood samples may be missed at some time points for some patients if their sampling times occur outside the working hours of the research team. In these instances, reliance is placed on the cooperation of ICU nursing staff for sample collection. When samples are missing, we will assume that they are missing at random (see Statistical Analysis) and will allow any participants with at least 1 sample to be included in the analysis.

### Dissemination

The ProMPT trial is a novel trial to assess the hypothesis that propofol supplementation of cardioplegia will give better myocardial protection. If this hypothesis is confirmed, we will design a larger trial to test the effect of propofol supplementation on clinical outcomes (eg, postoperative complications).

We have used broad eligibility criteria for the trial and expect the findings to apply to almost all patients undergoing standard CABG or AVR using CPB. The findings will be disseminated through usual academic channels (ie, presentation at international meetings, peer-reviewed publications, and through patient organizations and newsletters to patients, where available). Because propofol is widely available in the acute care setting and is relatively inexpensive, there should be few obstacles to adoption. We do not anticipate that the findings will be commercially exploitable.

## Results

Patients have been successfully recruited over a 2-year period. Results are to be published in late 2014.

## Discussion

### Contrary Evidence

Despite numerous reports supporting a cardioprotective effect of propofol during surgery [17-19,37], there is some evidence to the contrary. For example, studies comparing the volatile anesthetic sevoflurane with propofol for anesthesia during CABG surgery reported protection to be conferred by sevoflurane only [15,38]. This inconsistency may be explained in part by the chosen dose regimen because the cardioprotective effect of propofol has been shown to be dose dependent [19]. Clinical benefits appear to be more evident at higher doses [39] with a maintenance dose of approximately 4.2 μg/mL attenuating postoperative cellular damage and improving clinical outcome in patients undergoing CABG with CPB [40]. This dose has also been shown to confer significant cardioprotection against global ischemia in rats when used as an adjunct to warm or cold cardioplegia [30,32]. The underlying mechanism of protection is independent of any protection conferred by cardioplegia and hypothermia alone [41]. In addition, the intralipid emulsion used as a vehicle for propofol administration exhibits no protection against cardiac injury [32].

### Propofol Delivery and Stability in Cardioplegia Solution

One of the major challenges of this trial was to develop a method to deliver a specific dose of propofol/intralipid to the heart during surgery. Although delivering as an adjunct to cardioplegia was the most practicable approach, differences in the temperature and method of delivery of cardioplegia for CABG and AVR procedures had to be addressed. In addition, because propofol had not been used as an adjunct to cardioplegia in the human clinical setting before this trial, it was necessary to determine the occurrence and extent of any physical or physiochemical changes to propofol or intralipid when mixed with cardioplegia solution and 0.9% sodium chloride. The Fresenius Kabi Stability Assessment Unit at Cardiff University performed pH, visual, microscopic, and particle size distribution analysis of relevant combinations of samples at time points ranging from 0 to 24 hours. Evidence of instability was found in the propofol-containing samples after 8 hours. Therefore, a period of 6 hours was deemed a reasonable, safe period within which to use the trial solutions after they had been made.

### Supplementation Concentration

Propofol maintenance during CPB is common practice at the BHI. An infusion rate of 33 to 100 μg/kg/min (equating to 1.98-6 mg/kg/h) is used resulting in a circulating blood propofol concentration of between 1.3 and 3.6 μg/mL.

The trial has adopted a pragmatic approach, giving anesthetists the option to continue with this practice, resulting in baseline concentrations of up to 3.6 μg/mL of propofol for placebo arm participants. To ensure at least a doubling of the propofol concentration between placebo and intervention participants, anesthetists are asked to cap their usage of propofol during maintenance to a 3 μg/mL where possible. A 3 μg/mL blood propofol concentration is well under the dose required for clinical benefit [39]. Our supplementation concentration was set at 6 μg/mL to ensure that a potentially cardioprotective dose of between 6 and 9 μg/mL was achieved in patients allocated to the intervention arm.

The proposed maximum dose of 9 μg/mL does not exceed the level routinely observed in the circulation during induction of anesthesia for cardiac surgery (ie, a safe concentration to which the myocardium is routinely exposed).

Propofol clearance follows a 3-compartmental open model, with a first exponential phase half-life of approximately 1.6 to 4.0 minutes [42]. A short half-life and rapid plasma distribution of propofol results in fast onset and short duration of propofol action. Very little of the supplementary propofol remains in the circulation when the next dose of cardioplegia is administered. Hence, it is exceptionally unlikely that any AEs could be attributed to propofol supplementation postoperatively.

Designing a practicable method for delivering a potentially protective dose of propofol to the heart during cardiac surgery has been challenging, but recruitment has been steady and straightforward. If our approach confirms the potential of propofol to reduce damage to the heart during cardiac surgery, we will apply for funding to carry out a larger multicenter trial powered to detect a difference in a primary clinical outcome.

## Multimedia Appendix 1

Anesthetic protocol.

## Abbreviations

AE: adverse event
AVR: aortic valve replacement
BHI: Bristol Heart Institute
BRI: Bristol Royal Infirmary
CABG: coronary artery bypass grafting
CPB: cardiopulmonary bypass
CTEU: Clinical Trials and Evaluation Unit
GCP: Good Clinical Practice
HDU: high-dependency unit
ICU: intensive care unit
LV: left ventricular
MHRA: Medicines and Healthcare products Regulatory Agency
MLHF: Minnesota Living with Heart Failure
MPTP: mitochondrial permeability transition pore
NIHR: National Institute for Health Research
ProMPT: Propofol cardioplegia for Myocardial Protection Trial
REC: Research Ethics Committee
ROS: reactive oxygen species
SAE: serious adverse event
UKCRC: UK Clinical Research Collaboration
