# Supplementary material for: The Effects of Propofol Cardioplegia on Blood and Myocardial Biomarkers of Stress and Injury in Patients With Isolated Coronary Artery Bypass Grafting or Aortic Valve Replacement Using Cardiopulmonary Bypass: Protocol for a Single-Center Randomized Controlled Trial
Source: JMIR Res Protoc. 2014 Jul 8;3(3):e35. doi: 10.2196/resprot.3353 (PMC4115261; doi:10.2196/resprot.3353)
Supplement: Supplementary file 1 [file resprot_v3i3e35_app1.pdf]

ProMPT Anesthetic Protocol

Premed:

Temazepam 10-30 mg 1-2 hours before induction

Induction:

Midazolam; Fentanyl 5-10 mcg/kg; +/-Propofol up to 1 mg/kg

Muscle Relaxation as per standard practice

Maintenance Pre CPB:

Isoflurane plus boluses of Fentanyl as indicated (up to max 20 mcg/kg)

Maintenance on CPB:

Propofol TCI: initial target 3 mcg/mL (set according to the estimated ideal body weight) titrated up/down to response

Maintenance Post CPB:

Propofol TCI, converted to Propofol infusion (not target controlled) for transfer to CICU
